# Utilization of Intravenous Iron Therapy and Red Blood Cell Transfusion in Emergency Department Patients with Anemia: A Single-Center Retrospective Cohort Study

**DOI:** 10.3390/jcm15124552

**Published:** 2026-06-11

**Authors:** Sung-Joon Park, Min Joung Kim, Young-Hoon Yoon, Jung-Youn Kim

**Affiliations:** 1 Department of Emergency Medicine, College of Medicine, Korea University, Seoul 02841, Republic of Korea; 2 Department of Emergency Medicine, Korea University Guro Hospital, 148 Gurodong-ro, Guro-gu, Seoul 08308, Republic of Korea; 3 Department of Emergency Medicine, College of Medicine, Yonsei University, Seoul 03722, Republic of Korea

**Keywords:** emergency department, intravenous iron, red blood cell transfusion, ferric derisomaltose, length of stay, patient blood management

## Abstract

**Background/Objectives**: Anemia is frequently encountered in emergency departments (EDs). Although intravenous (IV) iron can be used as an alternative or adjunct to red blood cell (RBC) transfusion in selected hemodynamically stable patients, its use in the ED remains limited. This study described IV iron utilization and RBC transfusion patterns in ED patients with anemia and evaluated their associations with clinical outcomes. **Methods**: We conducted a single-center retrospective cohort study of patients who presented to a tertiary ED with hemoglobin (Hb) ≤ 10 g/dL between January 2019 and December 2021. Patients were categorized according to receipt of IV iron in the ED. Baseline characteristics, laboratory findings, transfusion practice, hospital length of stay (LOS), ICU admission, and in-hospital mortality were compared between groups. **Results**: Among 3340 patients, 89 (2.7%) received IV iron in the ED. IV iron recipients were older and had lower Hb levels than non-recipients. Gastrointestinal disorders were more frequent in the IV iron group (68.5% vs. 19.9%), and IV iron was commonly administered with ED RBC transfusion. ED transfusion (69.7% vs. 11.1%) and ICU admission (24.7% vs. 15.7%) were more frequent in the IV iron group. Among patients with available ferritin and transferrin saturation (TSAT), IV iron recipients had lower ferritin levels and more frequently showed ferritin-based or combined ferritin/TSAT findings suggestive of iron deficiency. In-hospital mortality was similar between groups (5.6% vs. 5.7%). Among hospitalized patients, median LOS was shorter in the IV iron group than in the non-IV iron group (6.6 vs. 9.7 days). **Conclusions**: IV iron was infrequently administered in ED patients with Hb ≤ 10 g/dL and was used mainly as an adjunct to RBC transfusion in older patients with gastrointestinal causes of anemia. Its association with shorter LOS should be interpreted cautiously. Structured ED-based anemia evaluation may help optimize IV iron use in selected patients.

## 1. Introduction

Anemia is a common hematologic disorder, affecting nearly 2 billion individuals globally, and contributing substantially to morbidity, impaired functional capacity, and increased healthcare utilization [1]. Iron deficiency anemia (IDA) accounts for approximately half of all anemia cases worldwide and represents the leading cause of this condition across all age groups and geographical regions [1,2,3]. Anemia is frequently encountered in the emergency department (ED) and may arise from a broad spectrum of etiologies including gastrointestinal hemorrhage, trauma, gynecological disorders, and chronic systemic diseases, each requiring tailored diagnostic and therapeutic approaches [4,5].

Historically, red blood cell (RBC) transfusion has been the predominant intervention for managing acute and chronic anemia in ED [4,6,7]. Although transfusion rapidly restores oxygen-carrying capacity, it is associated with infectious and noninfectious adverse events, including hemolytic and allergic reactions, transfusion-related acute lung injury (TRALI), and transfusion-associated circulatory overload (TACO) [8,9]. Circulatory overload risk is of particular concern in older patients and those receiving multiple transfusions [10]. Furthermore, inappropriate use of RBC transfusion imposes a significant financial burden and strains the already limited blood supply [4,11]. These concerns have led to increasing emphasis on patient blood management (PBM), which aims to optimize anemia treatment and reduce unnecessary allogeneic blood transfusion [11].

Intravenous (IV) iron therapy has emerged as a safe, effective, and cost-efficient alternative or adjunct to RBC transfusion in hemodynamically stable patients with IDA [4,12,13]. By rapidly replenishing iron stores, IV iron stimulates erythropoiesis and promotes sustainable hemoglobin recovery without the risks associated with allogeneic blood products [2,3,14]. Contemporary clinical practice guidelines and patient blood management recommendations emphasize restrictive RBC transfusion strategies and support iron replacement, particularly IV iron, as an important therapeutic option for hemodynamically stable patients with IDA [9,11].

However, IV iron remains markedly underutilized in ED settings [6,15,16]. Previous ED-based studies have reported similarly low IV iron utilization rates. In a Canadian ED study, IV iron was administered to only 4% of eligible patients, whereas 32% of RBC transfusions were deemed inappropriate [6]. In another study, the pre-protocol IV iron utilization rate was 4.4%, with concomitant excess transfusion volume [15]. These findings underscore the persistent knowledge-to-practice gap in anemia management in the ED. Dedicated quality improvement initiatives have shown that structured educational interventions and algorithmic approaches can substantially improve transfusion appropriateness and increase IV iron use [17].

To date, real-world data on IV iron utilization and its association with clinical outcomes in Korean ED settings are lacking. This retrospective cohort study aimed to characterize current RBC transfusion practices among ED patients with Hb ≤ 10 g/dL, to describe the utilization pattern of IV iron, and to evaluate its associations with transfusion volume, length of hospital stay, and in-hospital mortality.

## 2. Materials and Methods

### 2.1. Study Design and Setting

This single-center retrospective cohort study was conducted at a university-affiliated tertiary care hospital in South Korea. Patients who presented to the ED between 1 January 2019 and 31 December 2021, and had a documented Hb level ≤ 10 g/dL at the time of ED evaluation were included in the study population. Patients with incomplete medical records that precluded assessment of key study variables were excluded.

### 2.2. Data Collection

Baseline and clinical data were extracted from electronic medical records. Collected variables included: demographic information (sex and age); triage-level vital signs (systolic and diastolic blood pressure, heart rate, respiratory rate, body temperature, and peripheral oxygen saturation [SpO_2_]); ED triage acuity score; mode of arrival; chief complaint; primary ED diagnosis coded by International Classification of Diseases (ICD)-10; laboratory parameters obtained at ED presentation (hemoglobin [Hb], hematocrit [Hct], mean corpuscular volume [MCV], white blood cell count [WBC], platelet count, C-reactive protein [CRP], creatinine, albumin, and, when available, serum ferritin and transferrin saturation [TSAT]); the number of RBC units transfused in the ED and during subsequent hospitalization; administration and dosage of IV iron in the ED; ED disposition (admission, discharge, or transfer); ICU admission; hospital length of stay (LOS); and in-hospital mortality. The iron study results were summarized among patients with available ferritin and TSAT. LOS was calculated as the interval between ED arrival time and hospital discharge time and was restricted to admitted patients with complete discharge documentation.

### 2.3. Patient Grouping

Patients were stratified into two groups based on receipt of IV iron (Monofer^®^, ferric derisomaltose; Pharmacosmos A/S, Holbaek, Denmark) in the ED: the IV iron and non-IV iron groups. Baseline demographics, vital signs, laboratory parameters, disease category, ED and in-hospital transfusion volumes, LOS, ICU admission rates, and in-hospital mortality rates were compared between the two groups.

### 2.4. Statistical Analysis

All statistical analyses were performed using SPSS version 21.0 (IBM Corp., Armonk, NY, USA). Continuous variables with normal distribution are expressed as mean ± standard deviation (SD), whereas non-normally distributed continuous variables are expressed as median with interquartile range (IQR). Categorical variables are expressed as number and percentage. Between-group differences in normally distributed continuous variables were assessed using the independent samples *t*-test, whereas non-normally distributed continuous variables were compared using the Mann–Whitney U test. Categorical variables were analyzed using the chi-square test or Fisher’s exact test, as appropriate. Statistical significance was set at a two-tailed *p*-value < 0.05.

## 3. Results

### 3.1. Baseline Characteristics

**Figure 1:**
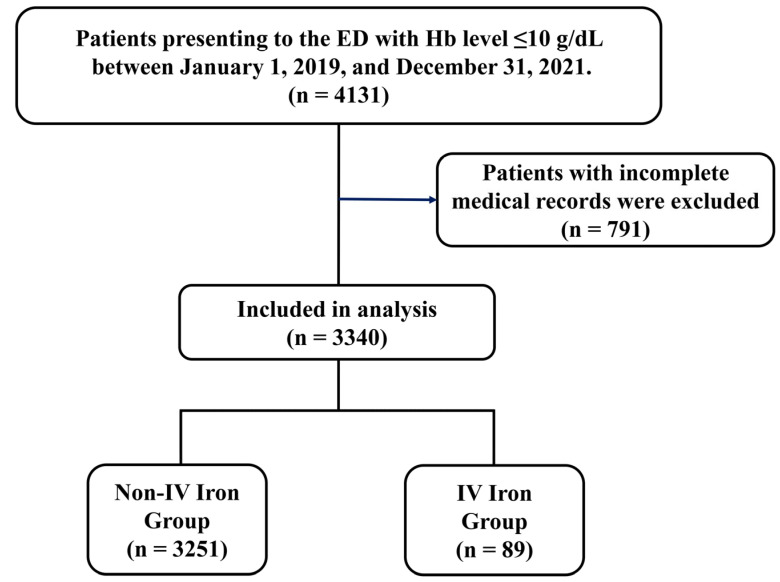
Flowchart of patient selection and study group allocation. ED, emergency department; Hb, hemoglobin; IV, intravenous.

A total of 3340 patients with Hb ≤ 10 g/dL were included in the final analysis, of whom 89 patients (2.7%) received IV iron in the ED. The flow of patient selection and study group allocation is shown in Figure 1. The overall cohort comprised 1740 females (52.1%) and 1600 males (47.9%), with a mean age of 61.3 ± 25.2 years. Patients who received IV iron were significantly older than those who did not receive IV iron (73.1 ± 13.3 vs. 61.0 ± 25.4 years, *p* < 0.001). When stratified by age group, no patients younger than 18 years received IV iron. Among IV iron recipients, 23 patients (25.8%) were aged 18–65 years, 36 (40.4%) were aged 65–80 years, and 30 (33.7%) were aged ≥ 80 years. Thus, 66 of 89 IV iron recipients (74.2%) were aged ≥65 years, suggesting that IV iron was preferentially used in older adults with anemia. Compared with the non-IV iron group, the IV iron group had lower Hb levels at ED presentation (8.08 ± 1.01 vs. 8.71 ± 1.02 g/dL, *p* < 0.001), lower hematocrit, lower MCV, and lower serum albumin levels. Although the mean CRP level was elevated in both groups, it was significantly lower in the IV iron group than in the non-IV iron group (31.5 ± 55.0 vs. 55.7 ± 79.7 mg/L, *p* = 0.005). WBC and platelet counts did not differ significantly between the groups. ED RBC transfusion, hospital admission, and ICU admission were more frequent in the IV iron group, whereas in-hospital mortality was similar between the two groups. Baseline characteristics, transfusion practice, and clinical outcomes are summarized in Table 1.

### 3.2. Disease Category Distribution

The most common disease category in the overall cohort was gastrointestinal disorders (709 patients, 21.2%), followed by trauma (478 patients, 14.3%), respiratory diseases (314 patients, 9.4%), genitourinary conditions (268 patients, 8.0%), infectious diseases (249 patients, 7.5%), and hematologic or oncologic disorders (246 patients, 7.4%). Gastrointestinal disorders were markedly more frequent in the IV iron group than in the non-IV iron group (68.5% vs. 19.9%), whereas trauma, respiratory, neurological, pediatric, and other disease categories were less frequent in the IV iron group (Table 2). Among the 61 IV iron recipients with gastrointestinal disorders, the detailed categories were non-variceal gastrointestinal bleeding in 37 patients (60.7%), gastrointestinal malignancy in 10 (16.4%), variceal bleeding in 7 (11.5%), hepatobiliary or pancreatic malignancy in 6 (9.8%), and inflammatory bowel disease in 1 (1.6%) (Supplementary Table S1). These findings indicate that IV iron was most commonly used in patients with gastrointestinal causes of anemia, including bleeding-related and malignancy-associated anemia. Infectious diseases accounted for 7 patients (7.9%) in the IV iron group and 242 patients (7.4%) in the non-IV iron group. Among the seven IV iron recipients categorized as having infectious diseases, no documented bacteremia or sepsis was identified.

### 3.3. Hemoglobin Level, MCV Category, and Iron Study Findings

Most patients had mild-to-moderate anemia at ED presentation. In the overall cohort, 1008 patients (30.2%) had Hb 8.1–9.0 g/dL and 1607 patients (48.1%) had Hb 9.1–10.0 g/dL. In the IV iron group, no patient had Hb ≤ 6.0 g/dL, whereas most IV iron recipients had Hb levels between 7.1 and 9.0 g/dL (67 of 89 patients, 75.3%) (Table 3). Among IV iron recipients, 27 patients (30.3%) had microcytic anemia, 59 (66.3%) had normocytic anemia, and 3 (3.4%) had macrocytic anemia. These findings indicate that IV iron administration was not limited to patients with microcytosis (Table 4). Iron study results were available in 563 patients with both ferritin and TSAT. In this subgroup, IV iron recipients had significantly lower ferritin levels than non-IV iron patients (60.3 [18.2–135.0] vs. 332.1 [112.1–708.2] ng/mL, *p* < 0.001). Ferritin < 100 ng/mL was more frequent in the IV iron group than in the non-IV iron group (62.1% vs. 24.4%, *p* < 0.001), as was ferritin < 100 ng/mL combined with TSAT < 20% (48.3% vs. 18.8%, *p* < 0.001). In contrast, TSAT < 20% alone did not differ significantly between groups (53.4% vs. 65.5%, *p* = 0.069) (Table 5).

### 3.4. Transfusion Practice and IV Iron Dosing

ED RBC transfusion was more frequent in the IV iron group than in the non-IV iron group. The mean number of RBC units transfused in the ED was higher in the IV iron group (1.74 ± 2.36 vs. 0.31 ± 1.10 units, *p* < 0.001), and the proportion of patients transfused in the ED was also higher (69.7% vs. 11.1%, *p* < 0.001). However, the number of RBC units transfused during hospitalization did not differ significantly between the groups (1.58 ± 2.02 vs. 1.13 ± 2.76 units, *p* = 0.129) (Table 1). All IV iron doses analyzed in this study were administered in the ED. During the study period, ferric derisomaltose was used in 200-mg vials. The most common dose was 1000 mg, administered to 48 patients (53.9%), followed by 600 mg in 28 (31.5%), 400 mg in 9 (10.1%), 200 mg in 3 (3.4%), and 1400 mg in 1 (1.1%) (Supplementary Table S2).

### 3.5. Clinical Outcomes

Hospital admission was more frequent in the IV iron group than in the non-IV iron group (97.8% vs. 73.7%, *p* < 0.001), whereas discharge from the ED was less frequent (2.2% vs. 24.9%, *p* < 0.001). ICU admission was also more common in the IV iron group (24.7% vs. 15.7%, *p* = 0.021). In-hospital mortality was similar between the two groups (5.6% vs. 5.7%, *p* = 0.977) (Table 1). Among hospitalized patients with complete discharge records, the median LOS was shorter in the IV iron group than in the non-IV iron group (6.6 [3.6–11.2] vs. 9.7 [5.1–18.5] days, *p* < 0.001).

## 4. Discussion

This retrospective cohort study examined the utilization of IV iron and RBC transfusion in 3340 ED patients with Hb ≤ 10 g/dL at a single tertiary care center in South Korea. The principal findings were as follows: (1) IV iron was administered to only 2.7% of the study cohort; (2) RBC transfusion was performed in 12.7% of patients during the ED visit; (3) IV iron recipients were predominantly older patients with gastrointestinal disorders and lower Hb levels; (4) IV iron was frequently used together with ED RBC transfusion rather than as a complete substitute for transfusion; and (5) IV iron administration was associated with a shorter hospital LOS among admitted patients, although this finding should be interpreted cautiously because of the retrospective study design and baseline differences between groups.

The low IV iron utilization rate observed in our cohort is consistent with previous ED-based studies and reflects a persistent gap in anemia management in the ED setting [6,15,16]. Previous studies have reported IV iron utilization rates of approximately 1.5–4.4% in ED patients with iron deficiency anemia or anemia-related presentations [6,15,16]. In our study, IV iron was administered to 2.7% of patients with Hb ≤ 10 g/dL. This rate should be interpreted as a real-world utilization rate within an ED anemia cohort, rather than as the proportion of all patients who were appropriate candidates for IV iron. Nevertheless, the low use of IV iron suggests that structured ED-based anemia evaluation and treatment pathways may be needed to better identify patients who could benefit from iron replacement [17,18,19,20].

Gastrointestinal disorders were markedly overrepresented among IV iron recipients. Detailed review of gastrointestinal disorders showed that most cases were related to non-variceal gastrointestinal bleeding, variceal bleeding, gastrointestinal malignancy, or hepatobiliary or pancreatic malignancy. These findings suggest that IV iron was most commonly used in patients with gastrointestinal causes of anemia, particularly bleeding-related or malignancy-associated anemia [15,19,20,21]. Importantly, the revised analysis of available iron studies provides additional context for this practice pattern. Among patients with available ferritin and TSAT, IV iron recipients had lower ferritin levels and were more likely to meet ferritin-based or combined ferritin/TSAT criteria suggestive of iron deficiency or iron-restricted erythropoiesis. However, because ferritin and TSAT were not systematically measured in all patients, confirmed IDA could not be established for the entire cohort.

MCV-based analysis also showed that IV iron administration was not limited to patients with microcytic anemia. Although 30.3% of IV iron recipients had microcytic anemia, most had normocytic anemia. This finding is clinically plausible in the ED setting because acute or subacute blood loss, inflammation, chronic disease, malignancy-associated anemia, or mixed etiologies may present with normocytic indices [4,5,12]. Therefore, MCV alone may not fully capture patients with suspected iron deficiency or blood loss-related anemia [4,5]. Nevertheless, MCV and iron studies are useful, readily available parameters that should be incorporated into standardized ED-based algorithms for anemia assessment and IV iron decision-making [6,17,18,19,20].

The IV iron group had higher rates of ED RBC transfusion, hospital admission, and ICU admission than the non-IV iron group. These findings suggest that IV iron was preferentially used in clinically selected patients with more complex presentations, rather than in lower-acuity anemia cases. The higher ED transfusion rate in the IV iron group does not indicate that IV iron replaced RBC transfusion in this cohort. Rather, IV iron appears to have been used mainly as an adjunct to transfusion in patients with lower Hb levels or gastrointestinal causes of anemia. This interpretation is important because IV iron does not provide immediate oxygen-carrying capacity and therefore cannot substitute for urgent RBC transfusion when rapid stabilization is required [7,12,21].

The elevated CRP level in the IV iron group also warrants cautious interpretation. CRP is a nonspecific inflammatory marker and may be elevated in patients with bleeding, malignancy, chronic inflammatory disease, or localized infection. In the present cohort, the mean CRP level was lower in the IV iron group than in the non-IV iron group, WBC count did not differ significantly between groups, and infectious disease categories were not more frequent among IV iron recipients. In addition, among IV iron recipients categorized as having infectious diseases, no documented bacteremia or sepsis was identified. These findings do not suggest that IV iron was preferentially administered to patients with severe systemic infection. However, IV iron should still be used cautiously in patients with active severe infection, and future protocols should explicitly address infection-related contraindications or precautions [12,13,18,20].

Among hospitalized patients with complete discharge records, the median LOS was shorter in the IV iron group than in the non-IV iron group. This observed association is notable but should not be interpreted as evidence of a direct treatment effect. Because this was not a randomized study, the LOS difference may have been influenced by disease category, admission indication, transfusion practice, clinical trajectory, physician decision-making, and unmeasured confounders. Previous ED-based anemia clinic and IV iron pathway studies have suggested that structured IV iron strategies may reduce transfusion exposure, hospitalization, ED LOS, or treatment-related costs in selected patients [15,18,19,20]. Accordingly, our findings should be regarded as hypothesis-generating. Prospective studies using standardized treatment algorithms and adjustment for disease severity are needed to determine whether ED-based IV iron administration can reduce transfusion requirements or shorten hospital stay [17,18,19,20].

From a patient blood management perspective, this study highlights the need for more systematic anemia evaluation in the ED. Appropriate use of IV iron may help reduce unnecessary transfusion and support sustainable hematologic recovery in selected hemodynamically stable patients with confirmed or suspected iron deficiency [6,12,15,17,18,19,20]. However, the decision to administer IV iron should be guided by clinical context, hemodynamic status, bleeding severity, iron studies, MCV, and potential contraindications [4,5,12,18,20]. ED-based protocols incorporating ferritin, TSAT, and MCV may improve patient selection and reduce variation in practice [6,17,18,19,20].

This study has several limitations. First, the retrospective single-center design limits generalizability and introduces potential selection bias. Patients who received IV iron were not randomly assigned, and unmeasured confounders may have influenced both the decision to administer IV iron and clinical outcomes. Second, the number of IV iron recipients was small, limiting the ability to perform robust subgroup analyses or causal inference. Third, Hb ≤ 10 g/dL was used to define the ED anemia cohort and should not be interpreted as indicating that all included patients were appropriate candidates for IV iron therapy. Fourth, iron status parameters, including ferritin and TSAT, were not systematically documented for all patients, precluding confirmation of IDA in the entire cohort and limiting assessment of guideline-concordant IV iron use. Fifth, thalassemia and other hemoglobinopathies were not systematically evaluated, although these conditions may be relevant when assessing the appropriateness of IV iron therapy. Sixth, the clinical rationale for IV iron prescription, including symptom burden, hemodynamic status, bleeding activity, and physician decision-making, could not be fully ascertained from medical records. Finally, long-term outcomes after discharge, including hemoglobin recovery, re-transfusion, recurrent ED visits, and adverse events, were not assessed. Future prospective studies incorporating structured IV iron protocols with pre- and post-implementation audits are needed to clarify the clinical and health system effects of ED-based IV iron strategies.

## 5. Conclusions

In this single-center retrospective cohort study of ED patients with Hb ≤ 10 g/dL, IV iron was infrequently administered and was used predominantly in older patients with gastrointestinal causes of anemia. IV iron was commonly administered together with RBC transfusion, suggesting that it was used mainly as an adjunctive treatment in clinically selected patients rather than as a complete substitute for transfusion. Among hospitalized patients, IV iron administration was associated with a shorter LOS; however, this finding should be interpreted cautiously because of the retrospective study design and potential confounding. These findings highlight the need for structured ED-based anemia evaluation and iron replacement strategies incorporating clinical context, MCV, ferritin, and TSAT. Further prospective studies are warranted to clarify the appropriate role and clinical impact of IV iron therapy in ED patients with anemia.

## Figures and Tables

**Table 1:** Baseline demographics, vital signs, laboratory findings, transfusion practice, and clinical outcomes stratified by IV iron administration.

Variable	Total (*N* = 3340)	IV Iron Group (*n* = 89)	Non-IV Iron Group (*n* = 3251)	*p*-Value
**Demographics**				
Female sex, *n* (%)	1740 (52.1%)	42 (47.2%)	1698 (52.2%)	0.348
Age (years), mean ± SD	61.3 ± 25.2	73.1 ± 13.3	61.0 ± 25.4	<0.001
<18 years, *n* (%)	324 (9.7%)	0 (0.0%)	324 (10.0%)	-
18–65 years, *n* (%)	1181 (35.4%)	23 (25.8%)	1158 (35.6%)	-
65–80 years, *n* (%)	861 (25.8%)	36 (40.4%)	825 (25.4%)	-
≥80 years, *n* (%)	974 (29.2%)	30 (33.7%)	944 (29.0%)	-
**Vital Signs at ED Presentation**				
Systolic BP (mmHg)	125.2 ± 26.7	122.7 ± 27.4	125.2 ± 26.7	0.421
Diastolic BP (mmHg)	74.5 ± 16.1	70.0 ± 14.3	74.6 ± 16.1	0.012
Respiratory rate (/min)	21.8 ± 5.0	21.3 ± 3.9	21.8 ± 5.0	0.332
Heart rate (/min)	95.5 ± 23.3	92.7 ± 18.8	95.5 ± 23.4	0.292
Body temperature (°C)	36.9 ± 0.9	36.7 ± 0.8	36.9 ± 0.9	0.005
SpO_2_ (%)	95.3 ± 6.5	96.2 ± 5.3	95.3 ± 6.6	0.320
**Laboratory Findings at ED Presentation**				
Hemoglobin (g/dL)	8.70 ± 1.03	8.08 ± 1.01	8.71 ± 1.02	<0.001
Hematocrit (%)	26.24 ± 5.31	24.38 ± 4.97	26.29 ± 5.32	<0.001
MCV (fL)	91.58 ± 10.09	87.09 ± 9.66	91.70 ± 10.07	<0.001
WBC (×10^3^/μL)	10.6 ± 14.3	9.0 ± 5.3	10.6 ± 14.5	0.309
Platelet count (×10^3^/μL)	223.1 ± 125.4	211.7 ± 123.1	223.4 ± 125.5	0.404
CRP (mg/L)	55.0 ± 79.2	31.5 ± 55.0	55.7 ± 79.7	0.005
Creatinine (mg/dL)	1.37 ± 1.81	1.61 ± 2.03	1.37 ± 1.81	0.218
Albumin (g/dL)	3.57 ± 0.58	3.27 ± 0.55	3.58 ± 0.58	<0.001
**Transfusion Practice**				
RBC units transfused in ED (mean ± SD)	0.35 ± 1.19	1.74 ± 2.36	0.31 ± 1.10	<0.001
Patients transfused in ED, *n* (%)	423 (12.7%)	62 (69.7%)	361 (11.1%)	<0.001
RBC units transfused during hospitalization (mean ± SD)	1.14 ± 2.79	1.58 ± 2.02	1.13 ± 2.76	0.129
**Clinical Outcomes**				
Hospital admission, *n* (%)	2482 (74.3%)	87 (97.8%)	2395 (73.7%)	<0.001
Discharge from ED, *n* (%)	810 (24.3%)	2 (2.2%)	808 (24.9%)	<0.001
ICU admission, *n* (%)	531 (15.9%)	22 (24.7%)	509 (15.7%)	0.021
In-hospital mortality, *n* (%)	190 (5.7%)	5 (5.6%)	185 (5.7%)	0.977
Length of hospital stay (days), median [IQR] †	9.5 [5.1–18.2]	6.6 [3.6–11.2]	9.7 [5.1–18.5]	<0.001

BP, blood pressure; CRP, C-reactive protein; ED, emergency department; ICU, intensive care unit; IQR, interquartile range; IV, intravenous; RBC, red blood cell; SD, standard deviation; WBC, white blood cell count. Continuous variables are presented as mean ± SD unless otherwise indicated. Categorical variables are presented as *n* (%). *p*-values were calculated using the independent samples *t*-test for continuous variables and the chi-square test or Fisher’s exact test for categorical variables, as appropriate, except where noted. † Calculated as the interval from ED arrival to hospital discharge; restricted to admitted patients with complete discharge records (*n* = 2478); *p*-value derived from the Mann–Whitney U test.

**Table 2:** Distribution of disease categories by IV iron administration status.

Disease Category	Total (*N* = 3340)	IV Iron Group (*n* = 89)	Non-IV Iron Group (*n* = 3251)
Gastrointestinal	709 (21.2%)	61 (68.5%)	648 (19.9%)
Trauma	478 (14.3%)	4 (4.5%)	474 (14.6%)
Respiratory	314 (9.4%)	3 (3.4%)	311 (9.6%)
Genitourinary	268 (8.0%)	4 (4.5%)	264 (8.1%)
Infectious disease	249 (7.5%)	7 (7.9%)	242 (7.4%)
Hematologic/Oncologic	246 (7.4%)	6 (6.7%)	240 (7.4%)
Neurological	240 (7.2%)	0 (0.0%)	240 (7.4%)
Pediatric	238 (7.1%)	0 (0.0%)	238 (7.3%)
Obstetric/Gynecologic	190 (5.7%)	0 (0.0%)	190 (5.8%)
Other	408 (12.2%)	4 (4.5%)	404 (12.4%)

Disease categories were classified based on the primary ICD-10 diagnosis recorded at ED presentation. ED, emergency department; ICD-10, International Classification of Diseases, 10th Revision; IV, intravenous.

**Table 3:** Distribution of admission hemoglobin levels by IV iron administration status.

Hb Range (g/dL)	Patients (*n*)	Proportion (%)	IV Iron Group (*n*)	Non-IV Iron Group (*n*)
≤6.0	48	1.4	0	48
6.1–7.0	159	4.8	8	151
7.1–8.0	518	15.5	29	489
8.1–9.0	1008	30.2	38	970
9.1–10.0	1607	48.1	14	1593
Total	3340	100.0	89	3251

Percentages were calculated using the total cohort as the denominator. Hb, hemoglobin; IV, intravenous.

**Table 4:** Distribution of MCV categories among IV iron recipients.

MCV Category	IV Iron Recipients (*N* = 89), *n* (%)	Hb (g/dL), Median [IQR]	MCV (fL), Median [IQR]
Microcytic anemia (<80 fL)	27 (30.3)	7.90 [6.20–8.90]	74.2 [69.0–78.1]
Normocytic anemia (80–100 fL)	59 (66.3)	8.70 [7.70–9.40]	91.8 [87.7–95.4]
Macrocytic anemia (>100 fL)	3 (3.4)	8.40 [6.80–9.30]	104.4 [102.2–108.7]

Data are presented only for patients who received IV iron in the ED. Hb and MCV values are presented as median [IQR]. ED, emergency department; Hb, hemoglobin; IQR, interquartile range; IV, intravenous; MCV, mean corpuscular volume.

**Table 5:** Iron study results among patients with available ferritin and TSAT.

Variable	Total (*N* = 563)	IV Iron (*n* = 58)	Non-IV Iron (*n* = 505)	*p*-Value
Ferritin, median [IQR]	319.8 [95.5–709.7]	60.3 [18.2–135.0]	332.1 [112.1–708.2]	<0.001
TSAT (%), median [IQR]	17.8 [9.6–37.0]	17.8 [5.5–25.8]	18.2 [15.5–47.7]	0.042
Ferritin < 30 ng/mL, *n* (%)	72 (12.8)	17 (29.3)	55 (10.9)	<0.001
Ferritin < 45 ng/mL, *n* (%)	99 (17.6)	27 (46.6)	72 (14.3)	<0.001
Ferritin < 100 ng/mL, *n* (%)	159 (28.2)	36 (62.1)	123 (24.4)	<0.001
TSAT < 20%, *n* (%)	362 (64.3)	31 (53.4)	331 (65.5)	0.069
Ferritin < 100 ng/mL and TSAT < 20%, *n* (%)	123 (21.8)	28 (48.3)	95 (18.8)	<0.001

Data are restricted to patients with available ferritin and TSAT results. Continuous variables are presented as median [IQR], and categorical variables are presented as *n* (%). *p*-values were calculated using the Mann–Whitney U test for continuous variables and the chi-square test or Fisher’s exact test for categorical variables, as appropriate. IQR, interquartile range; IV, intravenous; TSAT, transferrin saturation.

## Data Availability

The data presented in this paper are available upon reasonable request from the corresponding author. The data are not publicly available due to privacy and ethical restrictions.

## Supplementary Materials

The following supporting information can be downloaded at: https://www.mdpi.com/article/10.3390/jcm15124552/s1, Table S1: Detailed gastrointestinal disease categories among IV iron recipients; Table S2: Distribution of IV iron dose among ED IV iron recipients.
