# Supplementary material for: Utilization of Intravenous Iron Therapy and Red Blood Cell Transfusion in Emergency Department Patients with Anemia: A Single-Center Retrospective Cohort Study
Source: J Clin Med. 2026 Jun 11;15(12):4552. doi: 10.3390/jcm15124552 (PMC13302344; doi:10.3390/jcm15124552)
Supplement: Supplementary file 1 [file jcm-15-04552-s001.zip › jcm-4294716-supplementary.pdf]

Supplementary Materials

Supplementary Table S1. Detailed gastrointestinal disease categories among IV iron recipients

Disease category	Patients (N = 61), n (%)
Non-variceal gastrointestinal bleeding	37 (60.7)
Gastrointestinal malignancy	10 (16.4)
Variceal bleeding	7 (11.5)
Hepatobiliary or pancreatic malignancy	6 (9.8)
Inflammatory bowel disease	1 (1.6)

Detailed gastrointestinal disease categories were reviewed among IV iron recipients classified as having gastrointestinal disorders based on the primary ED diagnosis. ED, emergency department; IV, intravenous.

Supplementary Table S2. Distribution of IV iron dose among ED IV iron recipients

IV iron dose	Patients (N = 89), n (%)
200 mg	3 (3.4)
400 mg	9 (10.1)
600 mg	28 (31.5)
1,000 mg	48 (53.9)
1,400 mg	1 (1.1)

All IV iron doses were administered in the ED. During the study period, ferric derisomaltose was used in 200-mg vials. ED, emergency department; IV, intravenous.
